# Models of integration of TB and HIV services and factors associated with perceived quality of TB-HIV integrated service delivery in O. R Tambo District, South Africa

**DOI:** 10.1186/s12913-023-09748-2

**Published:** 2023-07-27

**Authors:** Ntandazo Dlatu, Benjamin Longo-Mbenza, Teke Apalata

**Affiliations:** 1 Division of Public Health, Department of Community Medicine, Faculty of Health Sciences, Walter Sisulu University, Mthatha, South Africa; 2 Division of Medical Microbiology, Department of Laboratory Medicine and Pathology, Faculty of Health Sciences, Walter Sisulu University and National Health Laboratory Services, Mthatha, South Africa

**Keywords:** Tuberculosis, HIV, TB/HIV integration, Healthcare services

## Abstract

**Background:**

Tuberculosis is the leading infectious cause of death among people living with HIV. Reducing morbidity and mortality from HIV-associated TB requires strong collaboration between TB and HIV services at all levels with fully integrated, people-centered models of care.

**Methods:**

This is a qualitative study design using principles of ethnography and the application of aggregate complexity theory. A total of 54 individual interviews with healthcare workers and patients took place in five primary healthcare facilities in the O.R. Tambo district. The participants were purposively selected until the data reached saturation point, and all interviews were tape-recorded. Quantitative analysis of qualitative data was used after coding ethnographic data, looking for emerging patterns, and counting the number of times a qualitative code occurred. A Likert scale was used to assess the perceived quality of TB/HIV integration. Regression models and canonical discriminant analyses were used to explore the associations between the perceived quality of TB and HIV integrated service delivery and independent predictors of interest using SPSS® version 23.0 (Chicago, IL) considering a type I error of 0.05.

**Results:**

Of the 54 participants, 39 (72.2%) reported that TB and HIV services were partially integrated while 15 (27.8%) participants reported that TB/HIV services were fully integrated. Using the Likert scale gradient, 23 (42.6%) participants perceived the quality of integrated TB/HIV services as poor while 13 (24.1%) and 18 (33.3%) perceived the quality of TB/HIV integrated services as moderate and excellent, respectively. Multiple linear regression analysis showed that access to healthcare services was significantly and independently associated with the perceived quality of integrated TB/HIV services following the equation: Y = 3.72–0.06X (adjusted R2 = 23%, p-value = 0.001). Canonical discriminant analysis (CDA) showed that in all 5 municipal facilities, long distances to healthcare facilities leading to reduced access to services were significantly more likely to be the most impeding factor, which is negatively influencing the perceived quality of integrated TB/HIV services, with functions’ coefficients ranging from 9.175 in Mhlontlo to 16.514 in KSD (Wilk’s Lambda = 0.750, p = 0.043).

**Conclusion:**

HIV and TB integration is inadequate with limited access to healthcare services. Full integration (one-stop-shop services) is recommended.

## Introduction

Even though tremendous progress has been made in the fight against the two epidemics over the years, tuberculosis (TB) still ranks as a major cause of mortality and poor health among people with HIV, especially in countries with low resources [1]. The spread of HIV in sub-Saharan Africa led to a sharp rise in the prevalence of tuberculosis in the region [2]. There is substantial evidence regarding the linkages between HIV and TB, with a higher likelihood of mortality among co-infected patients [3, 4]. Despite the high risk of HIV clients developing TB, programmes at the global, national, and local levels started largely with a vertical approach with little or no coordination [5–7]. This has resulted in poorly coordinated management of the two diseases, with damaging effects on clients and operational difficulties for service providers, especially in resource-limited settings [6]. As a response to the intensity of TB-HIV co-morbidity, the World Health Organization (WHO) proposed TB-HIV service integration at least at the facility level [8–10]. There is, however, no consensus regarding the form (whether partial or full) of integration or the levels at which integration should occur [8]. As a result, various models (linkage, collaboration, and full integration) have been implemented with several challenges across various settings [11–13]. For instance, in a linkage model, when a patient is diagnosed with either of the two infections, he/she is referred to another facility or unit to be tested for the other. The collaborated model is concerned with the partial integration of services, whereby a person who has been diagnosed through TB services will also be counselled and tested for HIV and then referred if positive. In a fully integrated model of care, all services for TB and HIV are provided in a single facility by the same service providers. An overwhelming body of evidence, however, suggests that the fully integrated option offers optimum benefits for clients, health systems, and workers [14–16]. Integration of tuberculosis and HIV services, particularly in resource-limited South African provinces such as the Eastern Cape Province, has been far from optimal despite the existence of policy frameworks for integration. Achieving widespread integration of TB-HIV care is still unsatisfactory, regardless of a documented intent toward full integration [17–22]. While this is not the first empirical discourse on TB-HIV integration in the country, this, to the best of our knowledge, marks the first attempt to investigate the operational challenges of TB-HIV integration from the perspectives of service providers and patients at the facility level. We used “complexity theory” as a theoretical framework to guide the conduct of this study [23].

## Materials and methods

**Fig. 1:**
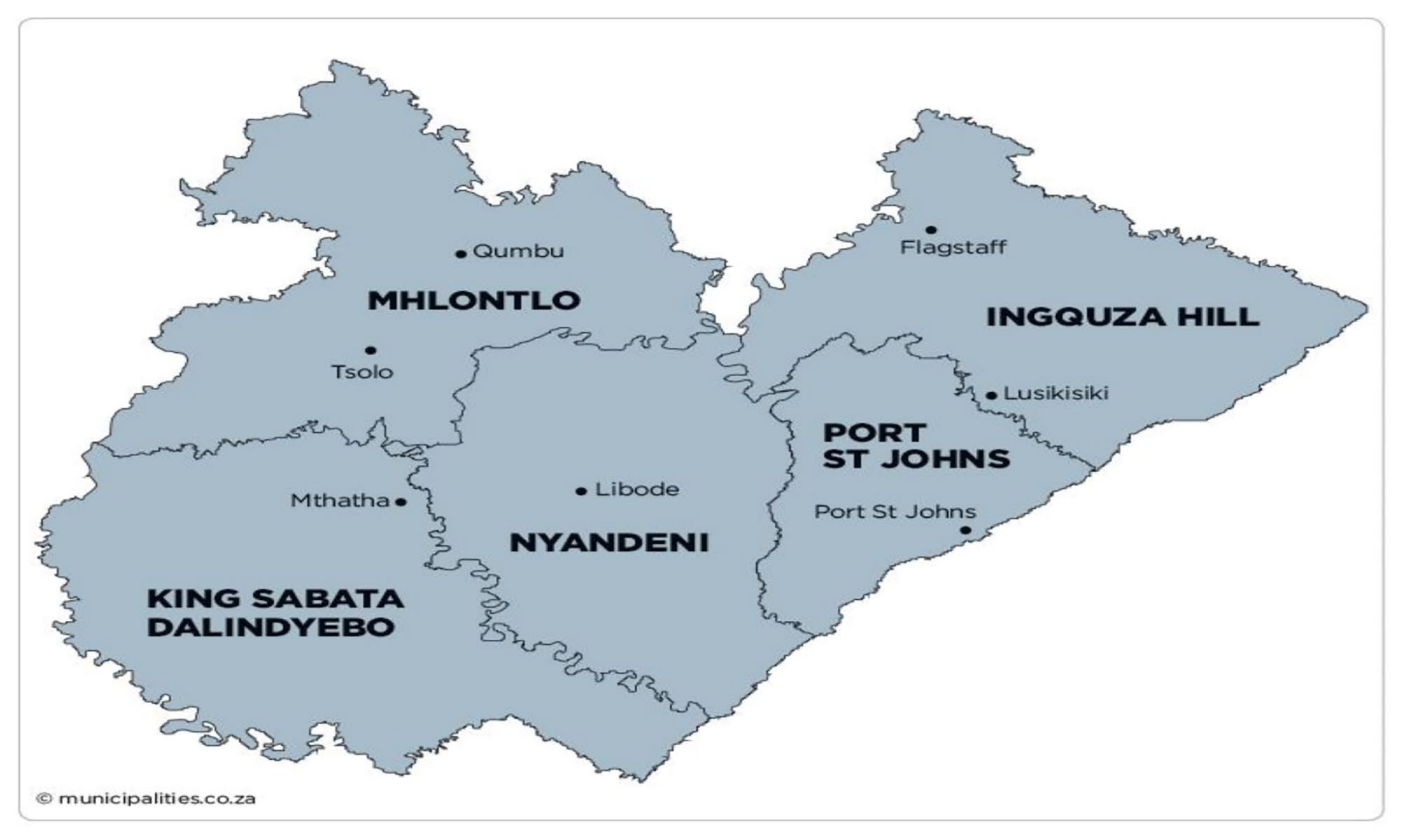
Map of O.R Tambo District Municipality. Source: http://isrdp.dplg.gov.za/documents/IDP/ISRDP/ OR_ Tambo_IDP.pdf. (open access)

O.R. Tambo district (see map in Fig. 1 below) is one of the 7 districts of the Eastern Cape province of South Africa. The seat of O.R. Tambo district is in Mthatha. The district has a total area of 12,141 km^2^, with a population estimated at 1,514,306 inhabitants at the time of the study. The racial makeup consists of Black-African 90.6%, colored 6.7%, Indian/Asian 1.2%, and white 1.0%, of which, based on language, Xhosa is the most dominant language spoken by 85% of people, followed by English 8.6% [24]. The district is made up of four health sub-districts: the King Sabata Dalindyebo (KSD) sub-district, the Mhlontlo sub-district, Nyandeni sub-district, and Qaukeni sub-district. While the four health sub-districts are deeply rural, the KSD sub-district is considered to be both rural and peri-urban. O.R. Tambo district has been reported to bear the following basic indicators: 64.6% of people are living in poverty, with an estimated unemployment rate of 65.5% and a literacy rate of 42.2%. The average annual income of a black resident is R15,762 [24].

### Description of the study areas

Health services are delivered by one central hospital, 1 regional hospital, 12 district hospitals; 11 community health centers, 49 clinics, 52 health posts, and 15 mobile health services. The main economic sectors include Community service (55%), trade (18.5%), finance (16.9%),agriculture (3.5%), transport (3.1%), manufacturing (2.8%) and construction (2.7%) [24].

### Study design, population, and data collection approach

This is a qualitative study design using principles of ethnography and the application of the aggregate complexity theory [25]. The researchers were trained by an expert in ethnographic studies before embarking on this study. This was critical for preparing the questionnaire and pretesting the questionnaire and conducting final interviews with participants.

**Fig. 2:**
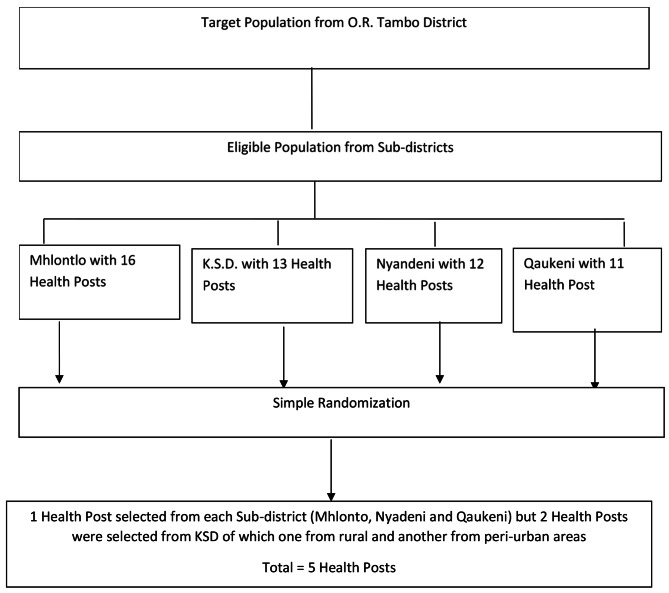
Flow chart representing the selection of study settings

Further ethnographic immersion involved the ability to converse with participants in their native language. As shown in the flow chart below (see Fig. 2), one health post per sub-district was selected following a simple randomization process, except for the KSD sub-district where 2 health posts were selected of which one from the deep rural setting and another one from the peri-urban setting. Although the 5 health posts were selected by simple randomization, they all have a relatively high burden of TB and HIV in their catchment areas.

Data for the study were drawn from a total of 25 health service providers and 27 TB/HIV patients from 5 selected health facilities across OR Tambo district. The participants were purposively selected based on their long-term direct involvement if they were patients or based on their working experience in the management of TB and or HIV in the selected facilities if they were clinic staff members. Although we can assume that staff members can have a different understanding of the problem than patients, we intentionally chose to combine patients and clinic staff members because, during an initial pilot study, we found that the vast majority of staff were not aware of the integrated TB and HIV policy and guidelines, and were not trained on their implementation. Technically, we hypothesized that these healthcare workers were not different from their patients when it came to TB/HIV integration policy and application. Given the focus of the study, a semi-structured interview guide was developed and used for the data collection. The interviews were based on one-on-one interaction with the participants and were all tape-recorded with their consent. This was done to ensure that we capture reality in the exact words of the individual participants. On average, each interview lasted 45 min.

### Theoretical framework: the complexity theory

**Fig. 3:**
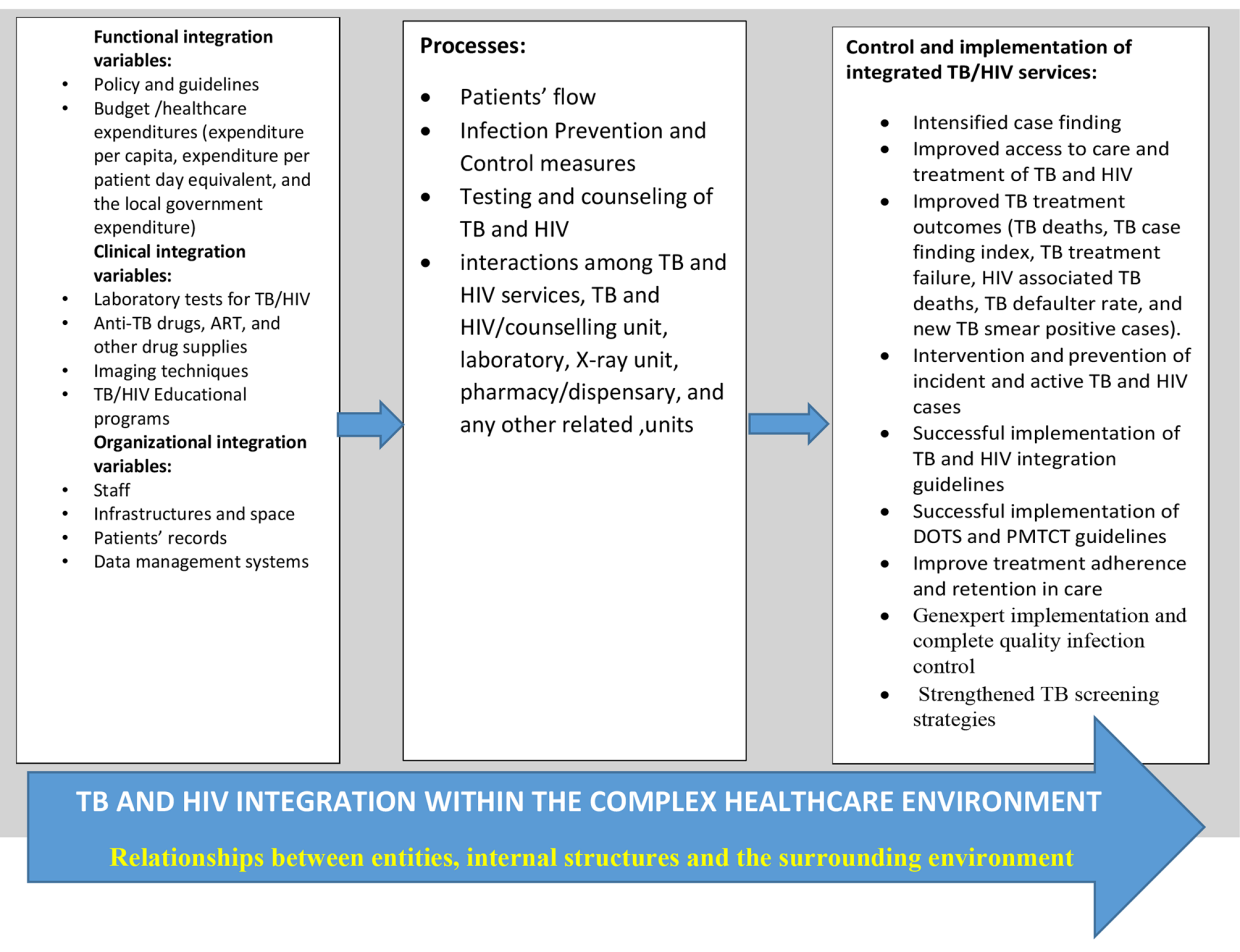
Theoretical framework: The complexity theory (Modified from Naidoo et al, 2017)

The underlying assumption in the complexity theory is the general agreement that healthcare delivery has become increasingly complex (see Fig. 3). This theoretical framework is mainly concerned with functionality and changes within a given healthcare system [25]. The same framework has been applied in other disciplines as well, but each of these disciplines provides a unique understanding of the concept. Manson (25) divided complexity theory into three; algorithmic complexity, deterministic complexity, and aggregate complexity [25].

In this study, we adopted the “aggregate complexity” because it considers the relationships between individual components in a complex adaptive healthcare system (i.e., integrated TB-HIV care operating within a wider healthcare system). Aggregate complexity is generally regarded as the qualitative component of the complexity theory [25]. Under this framework, it is estimated that all healthcare facilities, particularly those providing care for chronic diseases, are “complex adaptive systems”. The reason is that these healthcare facilities rely on individual agents, and specialized health workers in the field of TB and HIV management, whose actions are interconnected [26], and the interactions among the specialized units are more critical than the discrete actions of individual components [27]. In addition, the complexity theory considers the relationships between entities; internal structures and the surrounding environment; learning and emergent behaviour, and the various ways by which a complex system can transform and improve [28, 29]. During this study for instance, the concern is about the interactions among TB and HIV service providers, TB and HIV/counseling unit, laboratory, X-ray unit, pharmacy/dispensary, and any other units with a direct or indirect relationship with TB and HIV management and the clients. It is estimated that the actions of the related units should aim at ensuring a better outcome for the integrated healthcare services provided. Another factor to consider in this complex system is the environment, particularly the physical infrastructure for the management of TB and HIV in the health facility. The major strength of the aggregate complexity theory lies in its position that complex systems such as healthcare are constantly changing their internal structure and external environment. The complexity theory provides the researcher an opportunity to understand how a complex system like the integration of TB and HIV services can operate, and what are the impeding factors for scaling up the integration to produce the desired result.

### Data management and statistical analysis

At the end of each day of data collection, recorded interviews were transcribed and both old and newly emerging issues and themes through playback of the recorded interviews were noted. This was done to keep track of the issues in the data to identify issues that will require further probing in the subsequent interviews. Participants were enrolled until data reached saturation point, meaning that enough “Information Power” was achieved. Quantitative analysis of qualitative data was used. It “involved turning the data from words or images into numbers.

Quantification of qualitative data provided a dual benefit including the ease of analysis that quantitative data give us and the depth of meaning that qualitative data provided. When we wanted to quantify data regarding the estimated model of TB and HIV integration, the regression technique was used because from each qualitative answer from the respondents we were able to deduct a reference value of X, which means a model of TB/HIV integration. When we wanted to assess participants’ perceptions about TB and HIV integration, a probabilistic approach was used to quantify qualitative data by using different scoring techniques. Hence, the functional form of opinions about the relevant variable X was identified in the form of a score. In practical ways, quantification of qualitative data was done by coding ethnographic or other qualitative data, looking for emerging patterns, and counting the number of times a qualitative code occurs” [25]. When qualitative data was in the form of responses to standardized questionnaire surveys, this data was also quantified. In the end, the generated quantified data from coded qualitative data was used to create a single comprehensive dataset. Simple frequencies and associations between variables were then calculated. A Likert scale was composed of a series of four or more Likert-type items that represent similar questions combined into a single composite score (variable). Likert scale data were analyzed as interval data. For instance, means and standard deviations were used to describe the scale. In addition, the traditional way to report on a Likert scale was used meaning that the values of each selected option were summed and a score for each respondent was created. This score was then used to represent a specific trait-satisfied or dissatisfaction, for example. Another Scoring System consisted of (0 being poor and 6 being excellent). Regression models and canonical discriminant analysis (CDA) were used to explore the associations between the perceived quality of TB and HIV integrated service delivery and independent predictors of interest. During CDA, Fischer’s linear functions and Eigenvalues were determined with values of Wilk’s Lambda closer to zero being the evidence for well-discriminated patient groups. Data analysis was performed using SPSS®statistical software version 23.0 (Chicago, IL), considering a type I error of 0.05 as acceptable.

### Ethical approval and Consent to Participate

The Research Ethics and Biosafety Committee of the Walter Sisulu University approved the study **(ethical clearance No. 29/2014)** and permission to conduct the study was obtained from the Eastern Cape Department of Health **(EC_2016RP27_242).** The purpose and nature of the study were explained to the study participants and written consent was obtained individually from each participant. Participation was voluntarily, and confidentiality about their names and other identifiers were anonymously kept.

## Results

### Models of integration of TB and HIV Services

**Fig. 4:**
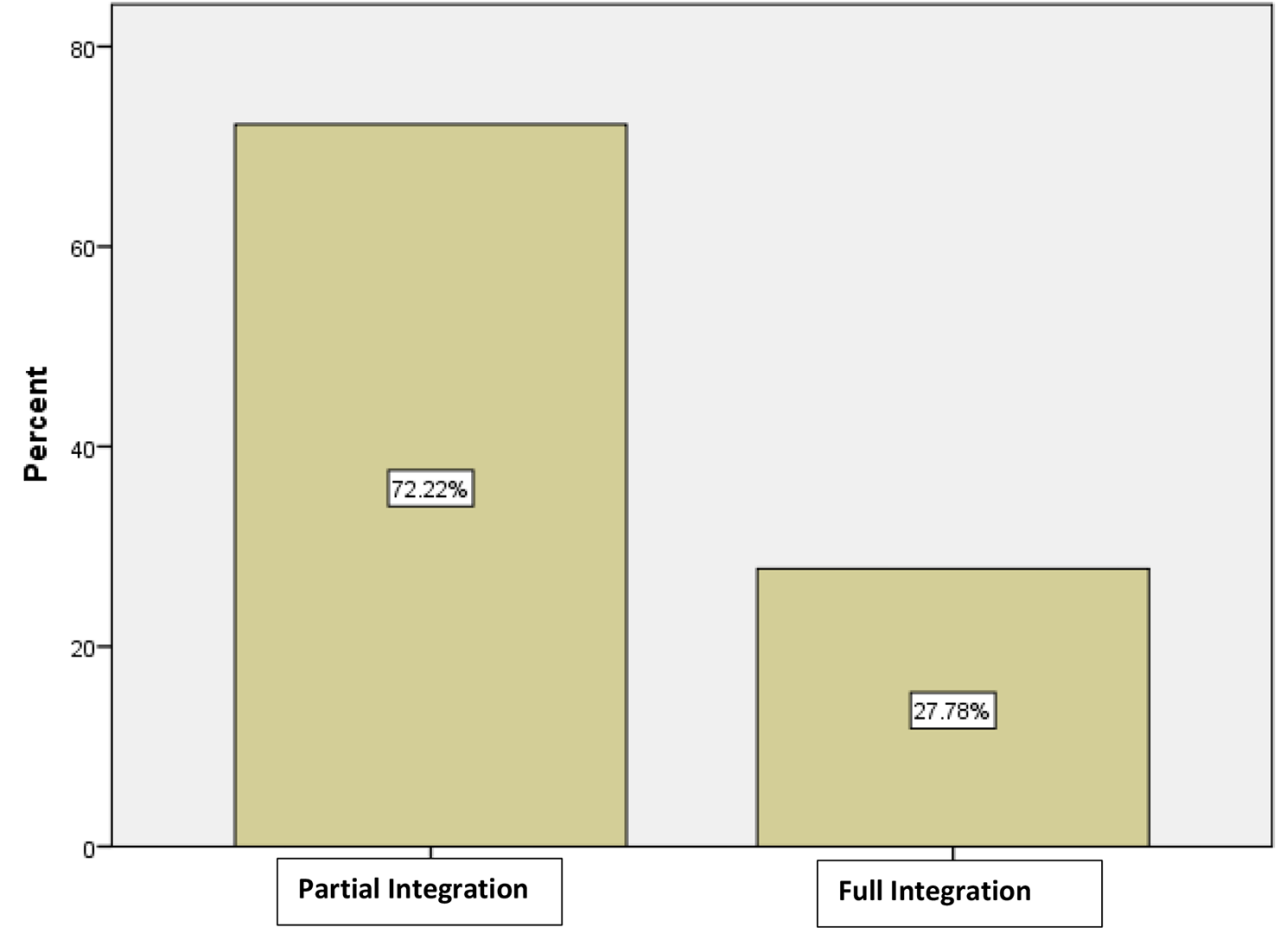
Models of Integration of TB and HIV Services in O.R Tambo district

Of the 54 participants, 39 (72.2%) reported that TB and HIV services were partially integrated while 15 (27.8%) participants reported that TB/HIV services were fully integrated as displayed in the Fig. 4, below.

**TB/HIV services were partially integrated when** tuberculosis and HIV/AIDS service delivery points are in the same health facility, but some HIV/AIDS services are provided in TB clinic, and some TB services are provided in HIV/AIDS clinic. The co-infected patients must still visit two different clinics served by different staff to access the full range of TB and HIV/AIDS services. However, **TB/HIV services were fully integrated when** TB and HIV/AIDS services are provided in the same delivery point in the health facility by the same staff (“One stop shop”).

**Perceived quality of services in relation to TB/HIV integration**.

**Fig. 5:**
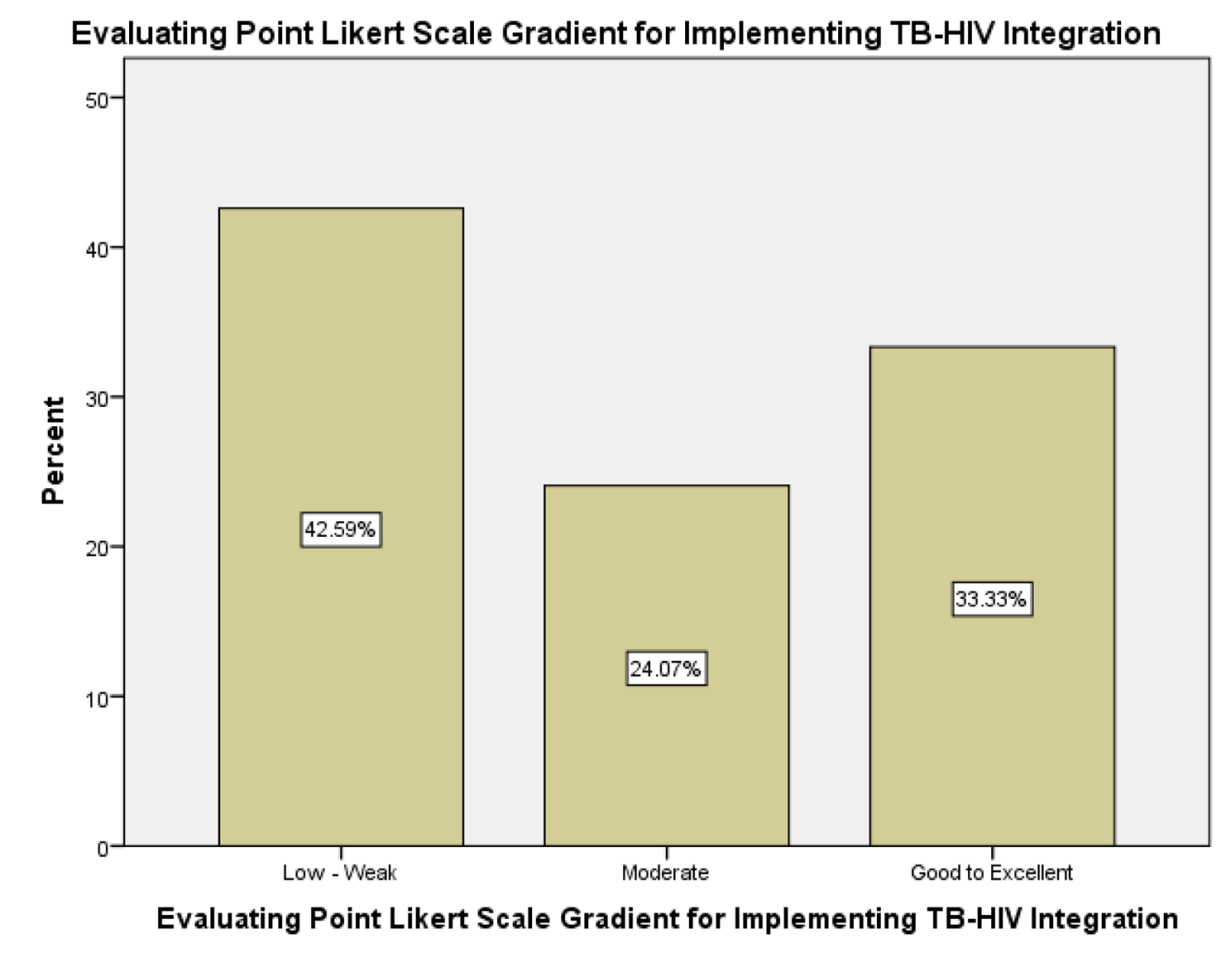
Perceived Quality of TB-HIV integrated Service Delivery in OR Tambo District using Likert scale gradient

Using Likert scale gradient, 23 (42.6%) participants perceived quality of integrated TB/HIV services as poor while 13 (24.1%) and 18 (33.3%) perceived quality of TB/HIV integrated services as moderate and excellent, respectively as display in the Fig. 5, below.

**Fig. 6:**
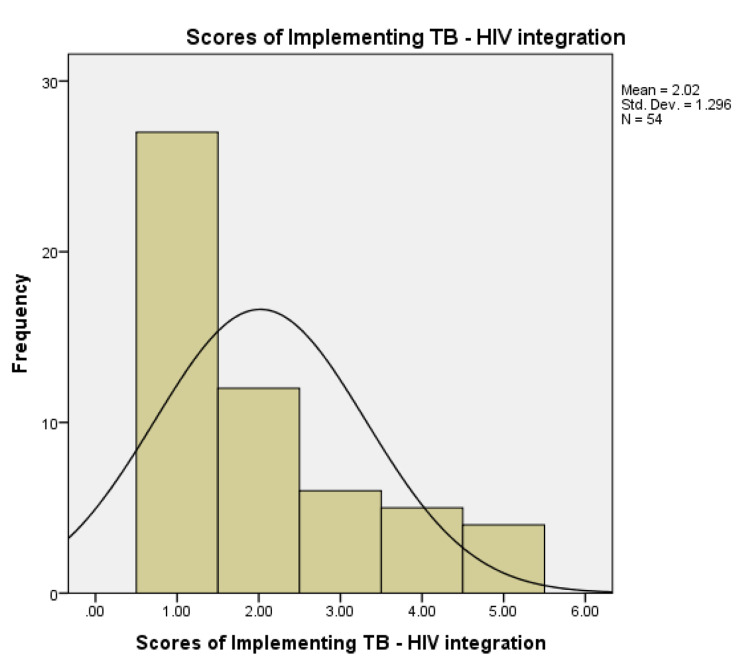
Perceived Quality of TB-HIV integrated Service Delivery in OR Tambo District using the Scoring System (0 being poor and 6 being excellent)

**Fig. 7:**
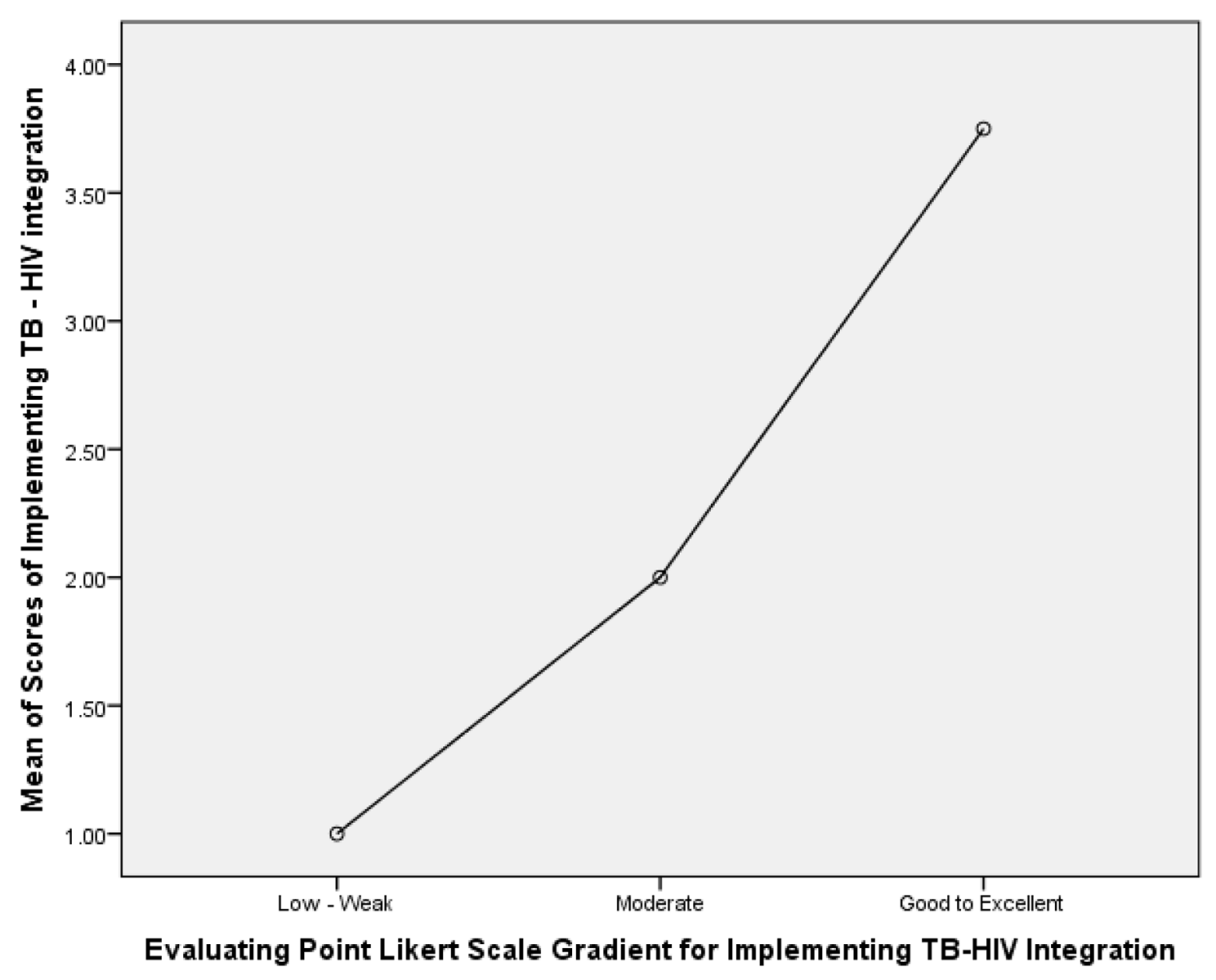
Relationship between Likert scale gradient and scoring system

When using the scoring system (ranging from 0 to 6 with 0 being poor and 6 being excellent) to measure the perceived quality of integrated TB/HIV services, the mean score for the 54 participants was found to be 2.02 (± 1.296 SD) with most participants scoring quality of services below 3 (P < 0.0001) as displayed **in** Fig. 6. The relationship between the Likert scale gradient and the scoring system is shown in Fig. 7.

### Perceived quality of integrated TB/HIV services by characteristics of the study participants

As shown in Table 1 below, participants perceived equally the integrated TB and HIV services as inadequate whether they were healthcare professionals or patients and whether they were males or females. However, marital status of the participants (p = < 0.0001), level of education of the HCWs (p = < 0.0001), participants’ home language (p = 0.049), and the municipality or health sub-district where the study was conducted (p = 0.003) have shown significant statistical differences on the participants perceived quality of TB/HIV integrated services.

**Table 1:** Association between Perceived quality of integrated TB/HIV services and characteristics of the study participants

Variables of interest	Perceived quality of integrated services as inadequate	Perceived quality of integrated services as adequate	p-value
**Category of participants**			**0.26**
Health professionals	16(64.0%)	9(36.0%)	
Patients	22(75.9%)	7(24.1%)	
**Gender**			**0.09**
Males	13(68.4%)	6(31.6%)	
Females	25(71.4%)	10(28.6%)	
**Living with**			**0.29**
alone	29(82.9%)	6(17.1%)	
spouse	9(47.4%)	10(52.6%)	
**Marital Status**			**< 0.0001**
Single	10(100.0%)	0(0.0%)	
Married	9(47.4%)	10(52.6%)	
Widowed	11(64.7%)	6(35.3%)	
Divorced	8(100.0%)	0(0.0%)	
**Qualification of HCWs**			**<0.0001**
High School	8(66.7%)	4(33.3%)	
Bachelor’s Degree	2(50.0%)	2(50.0%)	
Undergraduate Diploma	3(75.0%)	1(25.0%)	
Postgraduate Diploma	3(60.0%)	2(40.0%)	
**Population Group**			**0.08**
African	36(69.2%)	16(30.8%)	
White	2(100.0%)	0(0.0%)	
**Home Language**			**0.049**
English	3(100.0%)	0(0.0%)	
Xhosa	34(68.0%)	16(32.0%)	
Zulu	1(100.0%)	0(0.0%)	
Other	1(100.0%)	0(0.0%)	
**Province of upbringing**			**0.29**
Eastern Cape Province	37(69.8%)	16(30.2%)	
KwaZulu Natal Province	1(100.0%)	0(0.0%)	
**Working**			**0.32**
No	21(80.8%)	5(19.2%)	
Yes	17(60.7%)	11(39.3%)	
**Municipality**			**0.003**
KSD peri -urban Informal Mthatha Gateway	5(83.3%)	1(16.7%)	
Nyandeni small town Libode	5(38.5%)	8(61.5%)	
Mhlontlo - Mhlakulo small town	9(75.0%)	3(25.0%)	
Mhlontlo - Qumbu small town	8(66.7%)	4(33.3%)	
KSD Mbekweni rural areas	11(100.0%)	0(0.0%)	

Table 2 shows that participants from the KSD municipality (both rural and peri-urban) poorly perceived the quality of TB and HIV-integrated services. There was an inverse relationship between the age of the study participants and the perceived quality of integrated TB and HIV services. People living under higher poverty conditions perceived better quality of TB/HIV integrated care.

**Table 2:** Association between Participants’ perceptions of quality HIV/TB integrated services and local health municipality, age of participants, accessibility to healthcare facilities and poverty gap

Likert Scale Gradient	Variables of interest	Mean	Std. Deviation	P-value
**Low – Weak**				**< 0.0001**
	Age years	48.33	15.29	
	Access to services (Km)	29.89	6.25	
	Extent area (Km2)	2883.52	177.27	
	Poverty gap (%)	55.52	3.60	
**Moderate**				**0.001**
	Age years	44.55	15.17	
	Access to services (Km)	26.27	6.02	
	Extent area (Km2)	2816.18	192.87	
	Poverty gap (%)	56.23	4.59	
**Good to Excellent**				**0.001**
	Age years	41.25	14.52	
	Access to services (Km)	25.75	3.79	
	Extent area (Km2)	2661.19	201.45	
	Poverty gap (%)	59.78	5.91	

Multiple linear regression analysis showed that the access to healthcare services was significantly and independently associated with the perceived quality of integrated TB/HIV services following the equation: Y = 3.72–0.06X (adjusted R^2^ = 23%, p-value = 0.001).

Canonical discriminant analysis (CDA) (Tables 3 and 4) showed that in all 5 municipal facilities, long distances to healthcare facilities leading to reduced access to services were significantly more likely to be the most impeding factor which is negatively influencing the perceived quality of integrated TB/HIV services with functions’ coefficients ranging from 9.175 in Mhlontlo to 16.514 in KSD (Wilk’s Lambda = 0.750, p = 0.043).

**Table 3:** Wilks’ Lambda with test of functions and statistical significance from DA

Step		Tolerance	Min. Tolerance	Sig. of F to Enter	Wilks’ Lambda
0	Age years	1.000	1.000	0.328	0.957
	Access to services km	1.000	1.000	0.043	0.884
	Extent area km2	1.000	1.000	0.002	0.783
	Poverty Level %	1.000	1.000	0.016	0.850
1	Age years	0.840	0.840	0.941	0.781
	Access to services km	0.756	0.756	0.342	0.750
	Poverty Level %	0.248	0.248	0.669	0.770

**Table 4:** Classification of Function Coefficients on Municipality From: Fisher’s linear discriminant functions in O.R Tambo Municipality

	Municipality					
	KSD peri - urban Informal Mthatha Gateway	Nyandeni small town Libode	Mhlontlo - Mhlakulo small town	Mhlontlo - Qumbu small town	KSD Mbekweni rural areas	
Age years	0.373	0.272	0.274	0.257	0.420	
Access to services km	14.297	11.468	9.175	13.761	16.514	
(Constant)	-234.608	-150.362	-98.863	-212.858	-311.798	

## Discussion

This study was conducted to examine the integration of HIV/AIDS, Tuberculosis, and patients’ services into the general health care systems. The study revealed that neither health professional staff nor TB/HIV co-infected clients are satisfied with the current model, which presents several constraints to the vision of a continuum of care. O.R Tambo District Municipality has embarked on integrating TB and HIV services with the expected outcome that patients “have access to a continuum of care and support services for TB and HIV/AIDS diagnosis, in all health care facilities and community-based care services”. Our study found that the currently used model of care at O.R Tambo district health facilities is the partially integrated model, of patient-centered care and prevention. Traditionally, national AIDS- and TB-control programs have functioned separately and are mirrored by distinct service delivery structures with little coordination of HIV and TB services for individual patients [17]. To improve the diagnosis, treatment, and outcomes for patients with both diseases, the World Health Organization (WHO) developed a framework of strategic collaborative activities to be performed as part of the health sector response to control HIV infection-related TB. The collaborated model is concerned with the partial integration of services whereby a person who has been diagnosed through TB services will also be counseled and tested for HIV and then referred if positive [17]. WHO [17], proposed a fully integrated model of care, where all services for TB and HIV are provided in a single facility and by the same service providers. An overwhelming body of evidence, however, suggests that the fully integrated option offers optimum benefits for clients, health systems, and workers [17, 18]. The majority of countries have overwhelmingly used a partial model of integrated TB-HIV services [19]. This model refers patients to services providing HIV testing, with or without subsequent HIV care [26, 30]. It is the most common model of TB-HIV service integration in most highly TB-HIV prevalent countries and settings [27, 31, 32]. This model is appropriate at the hospital or health center level where TB and HIV services are both available but full integration is not possible [28, 33–37]. Studies in Malawi have shown achievements on increase in HIV testing of TB patients from 59 to 83%; in addition, cotrimoxazole preventive therapy (CPT) and antiretroviral (ART) provision to HIV–TB co-infected patients improved from 88 to 100% and 18–25%, respectively, when using partial model [30, 32, 38–44]. Similarly, in Kenya, there were higher percentages of HIV testing of TB patients and CPT and ART provision than the prior implementation of integration [45, 46].

Our study participants perceived the quality of TB and HIV/AIDS integrated services as inadequate in O.R Tambo District, however, the participating municipality has displayed statistical differences (Table 1). Integrating HIV and TB services (hereafter written HIV-TB services) is a key strategy in reducing TB-related deaths among people living with HIV as studies elsewhere reported [31, 47–50]. Study findings demonstrated associations between age, accessibility to services, and poverty gap which had a major influence on the perceived quality of TB and HIV integration. In trying to understand why age could influence participants’ perception of the quality of integrated TB/HIV services: participants in the study, who had a short distance to the facility, perceived TB and HIV services as integrated (young participants), while the majority of those participants staying far apart from the services or facility, perceived services as not integrated at all (adults’ participants). Studies by Doward et al. [51] demonstrated that patients aged 15–24 years were least likely to initiate ART and believe that services were integrated [52] while Kadia et al. (58) reported that age 36 years and more were associated with not initiating ART and were far from facilities reported that services were not integrated. Similarly, in many resource-limited settings in Africa, health systems are still struggling in achieving full integration of TB-HIV integration, regardless of documented intents towards full integration. Studies reported that the majority of adult patients believed that TB and HIV services were not fully integrated especially those who are staying far from the health facilities whilst those that are nearby believe that in their health facilities, TB and HIV are integrated [18]. In Zambia and Sudan, observational studies of TB patients reported geographical location (distance) also influences perception about TB-HIV integrated services with participants who are far from the health facilities reporting that services are not integrated [53, 54]. These studies concur with the WHO study which proposed that services must be available where people live and work to achieve greater accessibility and quality [16]. Overwhelming literature has highlighted that distance to the facilities still does not promote equity, accessibility, and availability of services to where people are and is perceived as not an integrated model [54–57]. The district facilities in low-income countries still struggle to achieve service excellence and greater accessibility of integrated TB and HIV because of the distance to the facilities [58]. Studies elsewhere have observed that the lack of access to treatment centers especially when patients had to be accompanied considering the high cost of living and transportation is a challenge [59, 60]. Integrated TB/HIV care, in which the same healthcare team provides services to patients with HIV-associated TB, offers potential advantages over standard approaches and quality care for both TB and HIV patients [51, 52, 61]. Studies elsewhere observed that poor patients can only accept services as they have no option since they are desperate and in need of healthcare services; due to their socioeconomic status and desperation, they will believe that TB and HIV services are integrated [53, 54, 58, 62, 63]. Literature demonstrated that there is a positive correlation between poverty and Mycobacterium tuberculosis, primarily through (1) its influence on living conditions, such as people living in overcrowded and poorly ventilated homes, (2) prolonged diagnostic delay, and (3) increased vulnerability due to malnutrition and/or HIV infection especially partial integrated model [58, 64–66].

TB-HIV integrated services exist when TB and HIV services are provided by the same trained healthcare provider at the same visit, a ‘one-stop service’, the TB clinic provides HIV treatment and the HIV clinic provides TB treatment [17]. This model is considered the most efficient and effective way to provide comprehensive TB–HIV services, and it is appropriate for settings with high TB and HIV prevalence [18]. Results in Malawi have shown an increase of 12% in HIV testing of TB patients, from 85 to 97%, as well as an increase of 19% in ART uptake, from 44 to 63% [19] by using this model. In addition, the integrated services were acceptable among patients. Similarly, in Kenya, the integration of HIV–TB services demonstrated improvements in HIV testing of TB patients and the provision of CPT and ART [17, 18]. Other studies demonstrated that integrated services in rural clinics can ensure a faster and sustained uptake of HIV-positive clients accessing treatment services and a much lower rate of client drop-out compared with larger specialized HIV/AIDS treatment hospitals. Studies linked this observation to the fact that integrated clinics offered a multiple range of services and HIV/AIDS clinic services were integrated into general consultation [18–20]. Studies elsewhere reported similar findings, where patients who visited integrated HIV Care (IHC) clinics were more likely to achieve viral suppression [67]. Proponents of the integrated HIV clinic model also talk of the potential for improvements in the quality of care provided to patients in integrated care clinics [66, 67]. Another study suggested that the quality of care is observably better in integrated care clinics when compared to stand-alone facilities (probably because the clinicians were also spending more time interacting with their clients) [67]. Also, there was a report on the improvement in the uptake of non-HIV services (testing for syphilis) when antenatal care (ANC) was integrated with HIV services [62]. Integration led to similar positive outcomes among TB patients and among diabetics in chronic care. Integration may also have a positive influence on healthcare workers and hospital operations because they are maybe better trained in both TB and HIV management [61]. A study by Charles et al. [68], in their report on the mid-term evaluation of the integrated management of adolescent and adult illness in Ethiopia, concluded that the integration of services at health facilities has been beneficial to both HIV-positive clients and hospital workers since HIV positive patients get TB services while health worker gets training on management of both diseases. They have highlighted significant improvements in hospital utilization and increased access of clients to quality HIV services. Hospital workers felt that they were better trained and motivated by what they perceived to be an increase in their credibility as clinical care providers in the eyes of the community [68]. It is possible that older patients who had other comorbidities that contributed to unfavorable outcomes through fully integrated management can turn to favorable treatment outcomes [52,62, 63].

### Limitations of the study

This study could have been done at a large scale but due to very limited funding and time, we had to choose the five clinics and their patients. The time constraint was also a limitation as these clinics and staff were very busy with their daily activities. When using probabilistic and regression techniques during the quantification of qualitative data, limitations include the fact that estimation can be flawed by multicollinearity and numerical convergence problems. Also, the treatment of polychotomous questions may be complicated. Despite these limitations, the combination of both probabilistic and regression approaches minimized some of these issues, and we capitalized on the use of discriminant analysis, a powerful research analysis tool, to investigate how variables of interest contributed to group separation, and to what degree.

## Conclusion and recommendations

Overall, participants are not happy with the current model and type of services rendered by their facilities in O.R Tambo district. They are coming from impoverished communities and not having social protection; majority of them on grant dependent and lack health insurance. After traveling long distances to reach the healthcare facilities, they cannot get optimal quality integrated TB/HIV services since the district is still implementing partial integration model of TB and HIV care. The study recommends that O.R Tambo should strive for service excellency to achieve the fully integrated TB and HIV services for continuum of care. TB and HIV services should be equally accessible, acceptable and reach every client in the district, young and old, irrespective of gender, race, and ethnicity.

## Data Availability

The data generated and/or analysed during this study is available from the corresponding author on reasonable request.

## Abbreviations

HIV: Human Immunodeficiency Virus
TB: Tuberculosis
O.R Tambo: Oliver Reginald Tambo
ART: Antiretroviral
CDA: Canonical Discriminant Analysis
CPT: Cotrimaxozole Preventive Therapy
IPT: Isoniazid Preventive Therapy
UNAIDS: Joint United Nations Programme on HIV/AIDS
KSD: King Sabata Dalindyebo
MDR TB: Multiple Drug Resistance
XDR TB: Extreme Drug Resistance
